# Tocotrienol Affects Oxidative Stress, Cholesterol Homeostasis and the Amyloidogenic Pathway in Neuroblastoma Cells: Consequences for Alzheimer’s Disease

**DOI:** 10.3390/ijms17111809

**Published:** 2016-10-29

**Authors:** Marcus O. W. Grimm, Liesa Regner, Janine Mett, Christoph P. Stahlmann, Pascal Schorr, Christopher Nelke, Olga Streidenberger, Hannah Stoetzel, Jakob Winkler, Shatha R. Zaidan, Andrea Thiel, Kristina Endres, Heike S. Grimm, Dietrich A. Volmer, Tobias Hartmann

**Affiliations:** 1 Experimental Neurology, Saarland University, Kirrberger Straße 1, 66421 Homburg/Saar, Germany; liesa.regner@uks.eu (L.R.); janine.mett@uks.eu (J.M.); christoph.stahlmann@uks.eu (C.P.S.); pascals91@gmail.com (P.S.); christophernelke@yahoo.de (C.N.); olga.streidenberger@uks.eu (O.S.); hannah.stoetzel@uks.eu (H.S.); Jakob.winkler@hotmail.de (J.W.); shatharamadhan@yahoo.com (S.R.Z.); andrea.thiel@uks.eu (A.T.); heike.grimm@uks.eu (H.S.G.); tobias.hartmann@mx.uni-saarland.de (T.H.); 2 Neurodegeneration and Neurobiology, Saarland University, Kirrberger Straße 1, 66421 Homburg/Saar, Germany; 3 Deutsches Institut für DemenzPrävention (DIDP), Saarland University, Kirrberger Straße 1, 66421 Homburg/Saar, Germany; 4 Institute of Bioanalytical Chemistry, Saarland University, 66123 Saarbrücken, Germany; dietrich.volmer@mx.uni-saarland.de; 5 Department of Psychiatry and Psychotherapy, Clinical Research Group, University Medical Centre Johannes Gutenberg-University Mainz, Untere Zahlbacher Straße 8, 55131 Mainz, Germany; Kristina.Endres@unimedizin-mainz.de

**Keywords:** tocotrienol, vitamin E, Alzheimer´s disease, amyloid-β, tocopherol, Aβ degradation, β-secretase, γ-secretase

## Abstract

One of the characteristics of Alzheimer´s disease (AD) is an increased amyloid load and an enhanced level of reactive oxidative species (ROS). Vitamin E has known beneficial neuroprotective effects, and previously, some studies suggested that vitamin E is associated with a reduced risk of AD due to its antioxidative properties. However, epidemiological studies and nutritional approaches of vitamin E treatment are controversial. Here, we investigate the effect of α-tocotrienol, which belongs to the group of vitamin E, on AD-relevant processes in neuronal cell lines. In line with the literature, α-tocotrienol reduced the ROS level in SH-SY5Y cells. In the presence of tocotrienols, cholesterol and cholesterol esters, which have been shown to be risk factors in AD, were decreased. Besides the unambiguous positive effects of tocotrienol, amyloid-β (Aβ) levels were increased accompanied by an increase in the activity of enzymes responsible for Aβ production. Proteins and gene expression of the secretases and their components remained unchanged, whereas tocotrienol accelerates enzyme activity in cell-free assays. Besides enhanced Aβ production, tocotrienols inhibited Aβ degradation in neuro 2a (N2a)-cells. Our results might help to understand the controversial findings of vitamin E studies and demonstrate that besides the known positive neuroprotective properties, tocotrienols also have negative characteristics with respect to AD.

## 1. Introduction

Alzheimer’s disease (AD) is a devastating neurodegenerative disorder and the most common cause of dementia in the elderly, pathologically characterized by neuritic plaques and intracellular neurofibrillary tangles (NFTs). Neuritic plaques are composed of aggregated amyloid-β (Aβ) peptides, generated by sequential proteolytic processing of the amyloid precursor protein (APP) by the aspartyl-proteases β- and γ-secretase, whereas hyperphosphorylated microtubule-associated protein tau is the main component of NFTs [1,2,3]. Although extracellular neuritic plaques consisting of aggregated Aβ peptides are the undisputable characteristic feature of AD, increasing evidence suggests that the oligomeric form of Aβ with up to 50 Aβ subunits represents the most toxic species of Aβ [4,5]. Several mechanisms of Aβ toxicity play a crucial role in AD, including cholinergic dysfunction, disruption of calcium homeostasis, inflammation, increased oxidative stress and mitochondrial dysfunction, finally resulting in neuronal/synaptic dysfunction and neuronal loss [6,7,8,9,10]. So far, increased age seems to be the most significant risk factor contributing to sporadic AD. Furthermore, carrying one or two copies of the apolipoprotein E (Apo E) ε4 alleles, one of the main apolipoproteins in the brain importing cholesterol into neurons via lipoprotein receptor-related protein, is a major risk factor for late-onset sporadic AD [11]. Hypercholesterolemia has also been proposed to be a risk factor in Alzheimer´s disease, especially for the amyloid pathology, which has been confirmed by several longitudinal, population-based studies, demonstrating that cholesterol is associated with AD in later lifespan [12]. Further studies support that cholesterol metabolism is linked to the susceptibility to AD. Diet-induced hypercholesterolemia in a transgenic mouse model for AD increased total Aβ levels, whereas cholesterol-lowering drugs reduced Aβ pathology in transgenic mice [13,14,15]. Rabbits fed with a cholesterol-rich diet showed a two-fold increase in β-amyloid concentration in the hippocampal formation [16]. Molecular and mechanistic evidence points towards a direct effect of cholesterol in AD pathogenesis by enhancing β- and γ-secretase-mediated Aβ production [17,18,19,20,21]. Several clinical trials associated statins, inhibiting the 3-hydroxy-3-methylglutaryl-CoA reductase (HMGCR) and, thus, cholesterol de novo synthesis, with a diminished risk to AD [22,23,24], whereas other clinical studies showed no differences [25,26,27]. However, cholesterol modulation might be an effective approach for reducing the risk of developing AD.

**Figure 1:**
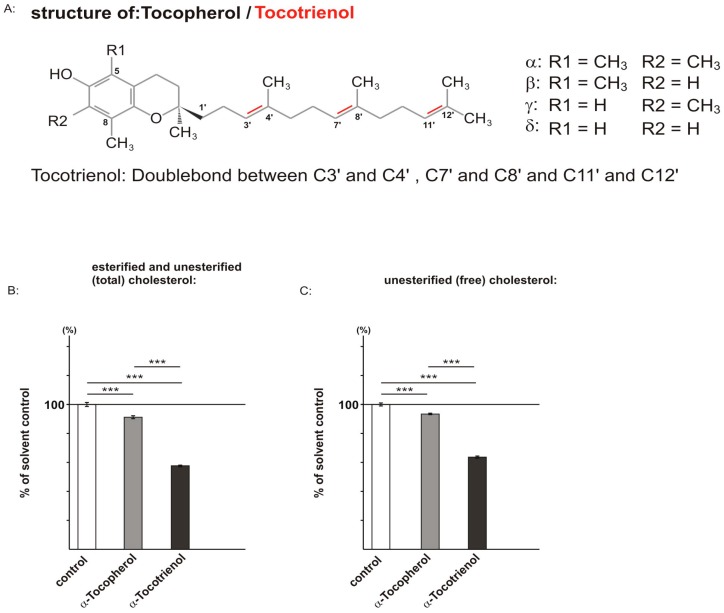
Effect of α-tocopherol and α-tocotrienol on cholesterol level. Error bars represent the standard deviation of the mean. Asterisks show the statistical significance calculated by post hoc ANOVA compared to the solvent control (*** *p* ≤ 0.001). (**A**) Skeletal formulas of α-tocopherol and α-tocotrienol. The red lines indicate the double bonds; (**B**,**C**) effect of α-tocopherol and α-tocotrienol in SH-SY5Y wildtype cells on esterified and unesterified (total) cholesterol (**B**) (α-tocopherol: 91.2% ± 1.0%, *p* ≤ 0.001, *n* = 8; α-tocotrienol: 57.7% ± 0.6%, *p* ≤ 0.001, *n* = 8) and unesterified (free) cholesterol (**C**) (α-tocopherol: 93.4% ± 0.5%, *p* ≤ 0.001, *n* = 8; α-tocotrienol: 63.7% ± 0.7%, *p* ≤ 0.001, *n* = 8) compared to the solvent control. One hundred percent in the control cells equates 0.0211 µg total cholesterol/µg protein (**B**) and 0.0161 µg free cholesterol/µg protein (**C**).

Molecules of the vitamin E family have been shown to exert hypocholesterolemic properties [28,29,30,31,32]. Vitamin E mainly consists of two families of lipophilic compounds: tocopherols and tocotrienols. Both families share a chromanol ring and an isoprenoid-derived side chain (Figure 1A). The side chain is saturated in tocopherols and unsaturated in tocotrienols. The chromanol ring of tocopherols and tocotrienols can be methylated at position C5 and C7 leading to α-, β-, γ- and δ-tocopherol or tocotrienol. Vitamin E is an essential component of the human diet and was primarily recognized for its antioxidant activity [33]. This micronutrient has already been proposed as a preventive agent in AD as increased oxidative stress by free radicals plays a crucial role in AD pathogenesis [34,35,36]. Tocotrienols also show a variety of neuroprotective activity, including anti-inflammatory activity [37,38,39,40,41]. However, besides the undisputable protective effects of vitamin E molecules, it has recently been shown that tocopherols increase the Aβ level [42]. However, so far, nothing is known about the amyloidogenic potential of tocotrienol. Therefore, we analyzed in the present study the effect of tocotrienol on Aβ generation and Aβ degradation.

## 2. Results

### 2.1. Effect of α-Tocopherol and α-Tocotrienol on Cholesterol Level

As described above, the vitamin E family consists of tocopherols and tocotrienols. α-tocopherol is the most abundant form among the tocopherols and mainly used in supplements; in accordance, α-tocotrienol is the most common form of the tocotrienols [43]. We therefore investigated the effect of both α-forms, which only differ in their chemical structure in the number of double bonds, on the cholesterol level.

Vitamin E compounds have been shown to exert hypocholesterolemic activity by affecting the sterol regulatory element binding protein/SREBP cleavage activating protein (SREBP/SCAP) system, one of the main systems regulating cholesterol homeostasis [28,30,44,45,46]. High cholesterol levels are discussed to be a risk factor for AD [12]. Cellular cholesterol exists in two major forms: (1) unesterified (free) cholesterol that possess a free 3-β-OH at the steroid ring B, instead of; and (2) cholesteryl esters (esterified cholesterol). Cholesterol acyltransferase (ACAT) responsible for the conversion of free cholesterol to esterified cholesterol has been shown to be affected in AD [47]. In principle, vitamin E molecules could not only influence cholesterol de novo synthesis by affecting the SREBP/SCAP system, but also alter cholesterol-esterase or cholesterol-ester synthase activity. Therefore, we determined in the present study unesterified, as well as total cholesterol levels.

As the above-mentioned studies were performed in non-neuronal cell lines, we used human neuroblastoma SH-SY5Y wildtype (wt) cells to determine the effect of α-tocopherol and α-tocotrienol on non-esterified and total cellular cholesterol, including esterified cholesterol level. Cells were maintained for three days in 1% fetal calf serum (FCS) in the presence of 10 µM α-tocopherol or α-tocotrienol, followed by treatment for 48 h in 0.1% FCS, to further reduce external sterol sources. Notably, we used a concentration of 10 µM, as it has been shown that tocopherol isomers occur in the human brain in concentrations of 0.11–17.9 µM [48]. Cholesterol levels were determined using an Amplex Red-based cholesterol assay, either in the absence of cholesterol-esterase for the determination of free cholesterol (unesterified cholesterol) or in the presence of cholesterol-esterase for the analysis of total cholesterol level (esterified and unesterified cholesterol). The α-tocopherol-treated cells showed a slight, but significant reduction in total cholesterol to 91.2% and free cholesterol level to 93.4% compared to solvent-treated SH-SY5Y wt cells (α-tocopherol: total cholesterol: 91.2% ± 1.0%, *p* ≤ 0.001; free cholesterol: 93.4% ± 0.5%, *p* ≤ 0.001) (Figure 1B,C). For α-tocotrienol, we found a strong and significant reduction in total and free cholesterol level. In the presence of α-tocotrienol, the total cholesterol level was decreased to 57.7% and the free cholesterol level to 63.7% (α-tocotrienol: total cholesterol: 57.7% ± 0.6%, *p* ≤ 0.001; free cholesterol: 63.7% ± 0.7%, *p* ≤ 0.001). Remarkably, compared to α-tocopherol, the reduction of total cholesterol and unesterified cholesterol level in α-tocotrienol-treated cells is highly significant (Figure 1B,C).

### 2.2. Reactive Oxidative Species Are Reduced in the Presence of α-Tocopherol and α-Tocotrienol

**Figure 2:**
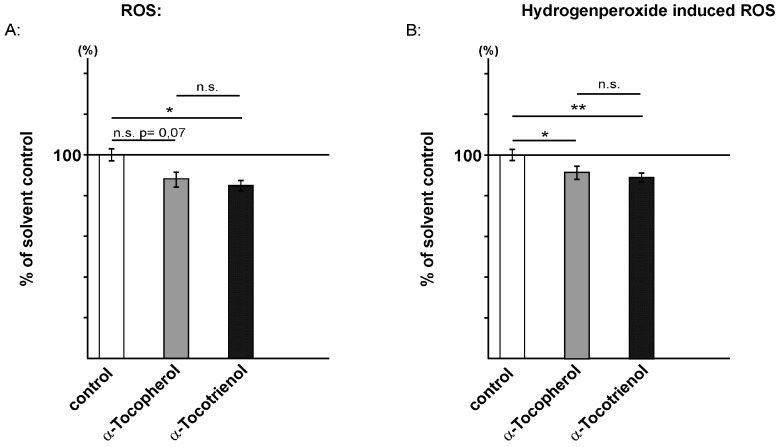
Effect of α-tocopherol and α-tocotrienol on oxidative stress. Reactive oxidative species measured with aminophenyl fluorescein (APF) (**A**) (α-tocopherol: 88.3% ± 3.6%, *p* = 0.07, *n* = 16; α-tocotrienol: 84.9% ± 2.6%, *p* = 0.012, *n* = 16) or induced reactive oxidative stress by hydrogen peroxide incubation (**B**) (α-tocopherol: 91.6% ± 3.2%; *p* = 0.029, *n* = 10; α-tocotrienol: 89.1% ± 2.6%, *p* = 0.006, *n* = 10) in the presence of α-tocopherol and α-tocotrienol in SH-SY5Y wildtype cells. Statistical significance as described for Figure 1 (* *p* ≤ 0.05, ** *p* ≤ 0.01 and n.s., not significant). In (**A**), the oxidative stress is measured by APF detecting several reactive oxidative species with different efficiency. Therefore, an absolute value of the ROS cannot be presented. However, we estimated the concentration of ROS by using NaOCl. The determined amount of NaOCl has shown the same APF-mediated fluorescence, which has been measured in the control cells. One hundred percent in the control cells equates to 2.89 nM NaOCl; In (**B**), 100% is the equivalent of 0.95 µM H_2_O_2_.

Vitamin E is known for its antioxidant activity [33]. To analyze whether tocopherols and tocotrienols exert different effects on the production of reactive oxidative species (ROS), we incubated SH-SY5Y wt cells 24 h in FCS-reduced culture medium in the presence of 10 µM α-tocopherol or α-tocotrienol. Notably, at these experimental conditions, α-tocopherol and α-tocotrienol showed no significant changes in cytotoxicity compared to solvent-treated control cells (Figure S1). 2-[6-(4’-amino)phenoxy-3*H*-xanthen-3-on-9-yl]benzoic acid (aminophenyl fluorescein (APF)) was used to selectively detect highly reactive oxygen species (hROS), including hypochlorite (–OCl) and free hydroxyl radical (·OH) [49]. The generation of ROS was slightly, but not significantly reduced to 88.3% in the presence of α-tocopherol (Figure 2A) compared to solvent-treated cells. A very similar, but significant reduction to 84.9% was obtained for α-tocotrienol (α-tocopherol: 88.3% ± 3.6%, *p* = 0.07; α-tocotrienol: 84.9% ± 2.6%, *p* = 0.012). The determination of ROS level, induced by using hydrogen peroxide (H_2_O_2_), revealed a significant reduction to 91.6% for α-tocopherol (α-tocopherol: 91.6% ± 3.2%; *p* = 0.029) (Figure 2B). The α-tocotrienol-treated cells showed a reduction to 89.1% for H_2_O_2_-induced ROS level (α-tocotrienol: 89.1% ± 2.3%, *p* = 0.006). Similar to the results for ROS level detected by APF, we observed no significant differences in H_2_O_2_-induced ROS levels between α-tocopherol and α-tocotrienol treatment (Figure 2A,B).

### 2.3. Effect of α-Tocotrienol on Aβ Generation and Aβ Degradation

**Figure 3:**
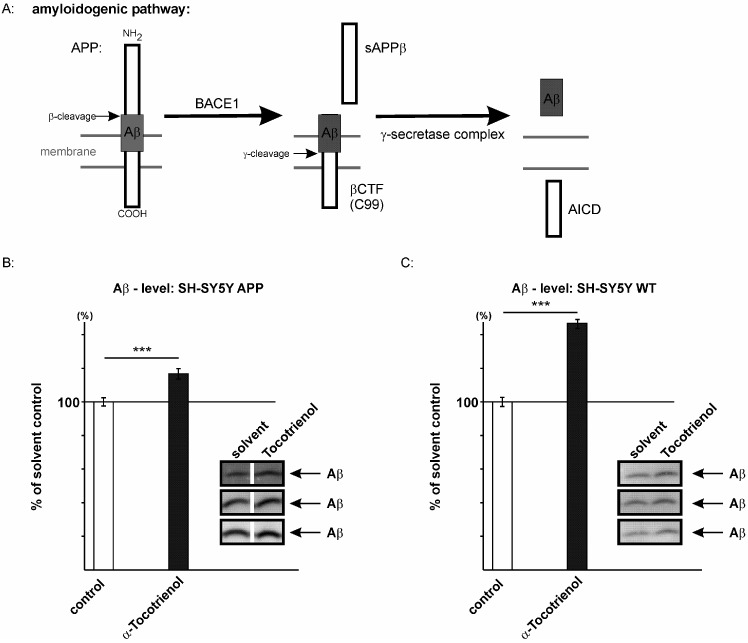

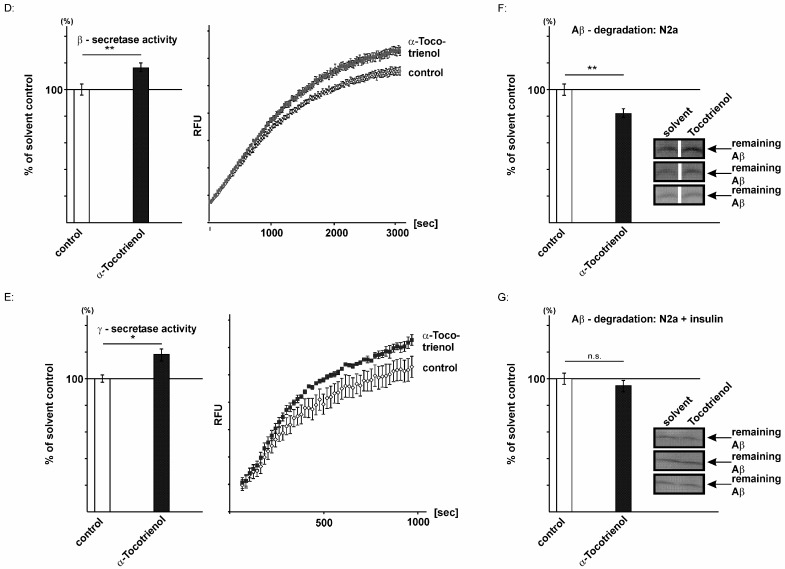
Effects of α-tocotrienol on Aβ-homeostasis. (**A**) Overview of the amyloidogenic pathway resulting in Aβ production; (**B**) enhanced secretion of Aβ by SH-SY5Y APP695 transfected cells after incubation with α-tocotrienol (116.7% ± 3.1%, *p* ≤ 0.001, *n* = 16). One hundred percent in the control cells corresponds to 8.9 ng/mL total Aβ; (**C**) elevated secretion of Aβ by SH-SY5Y wildtype cells after incubation with α-tocotrienol (146.7% ± 2.3%, *p* ≤ 0.001, *n* = 4). One hundred percent in the control cells equates to 3.1 ng/mL total Aβ; (**D**) β-secretase activity in purified membranes of SH-SY5Y cells treated with α-tocotrienol (116.3% ± 3.3%, *p* = 0.01, *n* = 6); (**E**) γ-secretase activity in purified membranes treated with α-tocotrienol of SH-SY5Y cells (118.3% ± 4.5%, *p* = 0.02, *n* = 6); (**F**,**G**) Effects of tocopherols on Aβ degradation; (**F**) increased protein level of non-degraded human Aβ or decreased Aβ degradation (82.1% ± 3.7%, *p* = 0.003, *n* = 6) in murine neuro 2a (N2a) cells incubated with α-tocotrienol compared to the solvent; (**G**) unaltered protein level of remaining human Aβ or unchanged Aβ degradation (98.8% ± 2.3%, *p* = 0.09, *n* = 6) in N2a cells incubated with 10 µM insulin in combination with α-tocotrienol or the solvent. Statistical significance was calculated using a two-tailed Student’s *t*-test (* *p* ≤ 0.05, ** *p* ≤ 0.01 and *** *p* ≤ 0.001, n.s., not significant).

In a previous study, we have shown that tocopherols increase the Aβ level [42]. The total Aβ level depends on Aβ generation by proteolytic processing of APP and Aβ clearance by Aβ degrading enzymes, like neprilysin or insulin-degrading enzyme (IDE) [50,51,52,53,54]. To analyze the effect of α-tocotrienol on Aβ generation, we focused on the amyloidogenic pathway involving β- and γ-secretase cleavage of APP (Figure 3A). β-site shedding of APP by BACE1 initiates the production of Aβ peptides, generating soluble sAPPβ and a membrane-tethered C-terminal fragment called βCTF or C99, which is further cleaved by the γ-secretase complex to release Aβ [2]. The influence of α-tocotrienol on the total Aβ level was analyzed in SH-SY5Y cells stably transfected with human APP695, an APP isoform mainly expressed in neurons [55]. The Aβ level was determined by using the antibody W02, recognizing an epitope between amino acids 1–10 of the Aβ peptide, thus, detecting the total Aβ level (Aβ1–X) including Aβ1–40 and Aβ1–42. α-tocotrienol significantly increased the total Aβ level to 116.7% (116.7% ± 3.1%, *p* ≤ 0.001) (Figure 3B). An increase in the Aβ level to 116.7% might appear rather small; however, one has to take into consideration that AD has a long preclinical phase and that a small increase in the Aβ level can result in an earlier disease onset [56].

To exclude unphysiologically-relevant effects by the use of APP overexpressing cells, we also incubated SH-SY5Y wildtype (wt) cells in the presence of 10 µM α-tocotrienol or the solvent control. For SH-SY5Y wt cells, we observed an even stronger increase in the Aβ level in the presence of α-tocotrienol (146.7% ± 2.3%, *p* ≤ 0.001) (Figure 3C). The increased effect strength might be explained by the fact that potential effects on Aβ degradation are less visible in a system where de novo synthesis of Aβ is as high as in APP overexpressing cells.

To analyze whether α-tocotrienol directly affects secretase activities, we used post-nuclear membrane fractions of SH-SY5Y wt cells and measured β- and γ-secretase activity. Therefore, purified membranes were prepared from SH-SY5Y wt cells. After preparation, these purified membranes were in vitro incubated in the presence or absence of α-tocotrienol, and β- and γ-secretase activity was determined. As α-tocotrienol was added after the membranes were prepared, the observed effects on secretase activities are independent of tocotrienol-mediated changes in gene expression or cholesterol level or other mechanisms, which are dependent on intact cells. We found that α-tocotrienol directly increased β-secretase activity to 116.3% (β-secretase activity: 116.3% ± 3.3%, *p* = 0.01) (Figure 3D). Western blot analysis of the sAPPβ level in the conditioned medium of tocotrienol-treated SH-SY5Y APP695 cells revealed an increased sAPPβ protein level to 136.8% (136.8% ± 3.3%, *p* = 0.004) (Figure S2), further underlining increased γ-secretase cleavage in the presence of tocotrienol. Similarly, we observed a direct effect of α-tocotrienol on γ-secretase activity, which was elevated to 118.3% in the presence of α-tocotrienol compared to solvent-treated control cells (γ-secretase activity: 118.3% ± 4.5%, *p* = 0.02) (Figure 3E). To verify the direct effect on γ-secretase activity, we investigated SH-SY5Y cells stably expressing C99. As the Aβ level in C99 transfected cells is only dependent on γ-secretase cleavage, potential alterations in the Aβ level are caused by changes in γ-secretase processing [57]. In line with the observed increased γ-secretase activity (Figure 3E), the Aβ level was elevated to 133.6% (133.6% ± 2.8%, *p* ≤ 0.001) in tocotrienol-incubated C99 transfected SH-SY5Y cells (Figure S3). The direct effect of tocotrienol on secretase activities could be substantiated by increased β- and γ-secretase activity in living SH-SY5Y wt cells in the presence of α-tocotrienol (β-secretase activity: 118.3% ± 4.6%, *p* = 0.001; γ-secretase activity: 132.6% ± 7.3%, *p* = 0.0003). Besides the direct effect of α-tocotrienol on β- and γ-secretase activity, we analyzed whether indirect tocotrienol-mediated mechanisms, like gene expression, might be involved in elevated amyloidogenic APP processing. Quantitative real-time PCR analysis revealed no changes in gene transcription of β-secretase BACE1 (Figure S4). Gene expression of the γ-secretase components presenilin 1 (PSEN1) and presenilin 2 (PSEN2), nicastrin (NCSTN), presenilin-enhancer 2 (PSENEN) and anterior-pharynx-defective 1A (APH1A) was also unchanged in the presence of α-tocotrienol (Figure S4). Only gene transcription of the γ-secretase component APH1B was slightly, but significantly reduced to 85.2% (85.2% ± 3.4%, *p* ≤ 0.001). In accordance with the unchanged gene transcription of BACE1 and nicastrin, the protein level of BACE1 and nicastrin was unaltered (Figure S5), further indicating that α-tocotrienol exerts its amyloidogenic potential by primarily directly affecting β- and γ-secretase. The overproduction of Aβ peptides, as well as reduced Aβ clearance contribute to AD [54,58]. Therefore, we analyzed whether α-tocotrienol affects Aβ degradation by incubating synthetic human Aβ40 in the presence of α-tocotrienol or the solvent on living cells for six hours and quantifying the remaining human Aβ40 by Western blot analysis. In order to avoid the detection of Aβ generated by the cells, we chose the mouse neuroblastoma cell line neuro 2a (N2a) and the antibody W02, recognizing human, but not mouse Aβ peptides, for this experiment (Figure 3F,G). In the presence of α-tocotrienol, Aβ degradation was reduced to 82.1% (82.1% ± 3.7%, *p* = 0.003) (Figure 3F), indicating that α-tocotrienol decreases Aβ degradation. Interestingly, in the presence of insulin, a competitive inhibitor of the IDE, one of the most important enzymes in Aβ degradation, tocotrienol, had no effect on Aβ degradation (93.8% ± 2.3%, *p* = 0.09) (Figure 3G). We therefore conclude that tocotrienol acts via a mechanism in Aβ degradation, in which IDE plays an essential role.

## 3. Discussion

Vitamin E is a natural phyto-compound and frequently used as a nutritional supplement. Tocopherols are mainly found in vegetable oil, whereas the second family of vitamin E, the tocotrienols, is sourced from cereal grains and palm oil [59]. Tocopherols and tocotrienols are discussed to be important for successful aging, including cognitive performance [60]. Besides the well-known antioxidative activity of vitamin E, molecules of the vitamin E family show neuroprotective, anti-inflammatory and cholesterol-lowering properties [28,33,40,41], mechanisms that are also important in the pathogenesis of AD [61]. Early studies already reported a hypocholesterolemic activity of synthetic and natural tocotrienols [28,30]. After the identification of the SREBP/SCAP system by the laboratory of Brown and Goldstein [62,63], more recent studies showed that members of the vitamin E family decrease the cellular cholesterol level by affecting the SREBP/SCAP system [44,45,46]. The SREBP/SCAP system is one of the main systems controlling the cellular cholesterol level. Briefly, when the cholesterol level is low, SREBP-2 (sterol regulatory element binding protein 2), which is tethered to the endoplasmic reticulum (ER) as a full-length protein, is escorted by SCAP (SREBP-2 cleavage activating protein) to the Golgi apparatus, where SREBP-2 is cleaved by site-1- and site-2-protease releasing the active N-terminal portion of SREBP-2, which migrates to the nucleus and upregulates genes involved in cholesterol synthesis and cholesterol uptake [62]. The resulting increased cholesterol level promotes binding of SCAP to Insig (insulin-induced gene), retaining full-length SREBP-2 in the ER. Song and DeBose-Boyd reported that δ-tocotrienol blocks processing of SREBP-2 and stimulates HMGCR degradation, whereas γ-tocotrienol enhances primarily HMGCR degradation in SV589 cells, an immortalized line of human fibroblasts expressing the SV-40 large T-antigen [44]. α-tocotrienol and all tocopherols showed no measurable effect on reductase degradation or SREBP processing [44]. However, α-tocopherol has also been reported to decrease activated nuclear SREBP-2 resulting in a reduction of the expression of 10 genes involved in cholesterol de novo synthesis [45]. In line with this study, we found significantly decreased cholesterol level in the presence of α-tocopherol in SH-SY5Y neuroblastoma cells: unesterified cholesterol was reduced to 93.4% and total cholesterol to 91.2%. α-tocotrienol treatment in human neuroblastoma cells resulted in an even more pronounced reduction in unesterified cholesterol and total cholesterol to 63.7% and 57.7%, respectively. Notably, the reduction of total cholesterol and unesterified cholesterol level in α-tocotrienol-treated cells was highly significant compared to α-tocopherol, indicating that α-tocotrienol has with respect to cholesterol and AD a more beneficial effect than α-tocopherol. In line with our study, Krycer et al. [46] reported decreased SREBP-2 activity in the presence of 10 µM α-tocotrienol by degrading mature SREBP-2. δ- and γ-tocotrienols were even more potent inhibitors of SREBP-2 than α-tocotrienol [46]. This trend was also observed in the study by Song and DeBose-Boyd [44].

According to the cholesterol-lowering properties of tocopherols and tocotrienols, several studies reported a lower risk of mild cognitive impairment (MCI) and AD in populations with elevated plasma levels of tocopherols and tocotrienols [64,65,66,67]. High dietary intake of the combination of all natural vitamin E congeners has been shown to exhibit protection against AD [68]. Dietary intake of mixed tocotrienols in subjects with cardiovascular risk factors and white matter lesions (WMLs) also revealed neuroprotective properties by reducing the progression of WMLs [69]. However, supplementing AD patients with vitamin E showed no clear beneficial effect on disease progress [70,71,72]. Meta-analysis of 135,967 participants in 19 clinical trials even revealed that high-doses (≥400 IU/d) of vitamin E supplement may increase all-cause mortality [73]. Despite the observed cholesterol-lowering effect of α-tocotrienol to approximately 60%, we found an increase of the Aβ level in α-tocotrienol APP695 transfected SH-SY5Y cells to 116.7%. Considering the cholesterol-lowering properties of α-tocotrienol, one would expect that total Aβ level should decrease, as cholesterol has been shown to increase Aβ level [17,18,20]. However, we found that α-tocotrienol has, besides the effect on cholesterol, a direct increasing effect on the secretases activities, resulting in an increased Aβ production. Under the conditions used in this study, the Aβ increasing effect of tocotrienol on the secretases was more pronounced than the Aβ lowering effect of a reduced cholesterol level. However, we cannot rule out that under different experimental conditions, the cholesterol lowering effect is the more dominant effect on the Aβ level.

The total Aβ level is increased by the direct effect of α-tocotrienol on β- and γ-secretase activity, as cell-free assays showed an increase in β-secretase activity to 116.3% and an elevation to 118.3% for γ-secretase, whereas gene transcription and protein level of β-secretase BACE1 and the components of the γ-secretase complex were unchanged. Besides directly affecting secretase activities, we found that α-tocotrienol decreased Aβ degradation to 82.1%, also resulting in elevated Aβ level. The inauspicious amyloidogenic potential of vitamin E molecules accompanied by a reduction in Aβ degradation was already shown in our previous study for tocopherols [42]. However, as vitamin E molecules exert different physiological activities, one cannot exclude that other indirect effects may also contribute to an increase in the Aβ level. Although vitamin E molecules function as an antioxidant, scavenging toxic free radicals discussed to contribute to the pathological processes of AD, one has to take into consideration that elevated Aβ levels in presence of tocopherols and tocotrienols might increase Aβ-induced oxidative stress [61,74]. Based on the multifaceted cellular mode of action of vitamin E, one might speculate that some patients might profit from vitamin E supplementation, but others not. For example, individuals with hypercholesterolemia might profit from vitamin E because of the cholesterol-lowering effect of vitamin E, whereas in individuals with normal or low cholesterol, the amyloid potential of tocopherols and tocotrienols predominates. On the other hand, individuals with a low plasma level of tocopherols and tocotrienols might also benefit from vitamin E supplementation. Nishida et al. have shown that depletion of vitamin E by crossing AD transgenic APP-Swedish mice with α-tocopherol transfer protein knock-out mice increased Aβ deposits in the brain, which was ameliorated with α-tocopherol supplementation [75]. In line with these findings, dietary supplementation with *N*-acetylcysteine, α-lipoic acid and α-tocopherol attenuated age-related alterations in amyloid β metabolism, an increase in APP, β-secretase activity and a decrease in the Aβ degrading enzyme neprilysin, in aged rat brain [76] and prevented deficits in learning and memory functions [77]. On the other hand, treating MCI/AD patients with vitamin E revealed no significant differences in the rate of progression to AD between the vitamin E and placebo groups [70,71].

## 4. Materials and Methods

### 4.1. Chemicals

Chemicals including (+)-α-tocopherol and (+)-α-tocotrienol, as well as cell culture media were purchased from Sigma (Taufkirchen, Germany) unless otherwise noted.

### 4.2. Cell Culture

SH-SY5Y wildtype (wt) cells were cultivated in Dulbecco’s Modified Eagle’s Medium (DMEM) containing 10% fetal calf serum (FCS) (PAN Biotech, Aidenbach, Germany). For SH-SY5Y APP (overexpressing the human APP695 isoform) and SH-SY5Y C99 cells (overexpressing the β-cleaved C-terminal fragment) [78], Hygromycin B (PAN Biotech) was supplemented in a final concentration of 0.3 mg/mL.

Neuro 2a (N2a) cells were cultivated in DMEM comprising 10% FCS, 1% penicillin/streptomycin solution, 2 mM l-glutamine, 0.1 mM MEM and 1 mM sodium-pyruvate.

Twelve hours before incubation, FCS in cell culture medium was reduced to 0.1%.

Tocopherol and tocotrienol (dissolved in ethanol) were incubated in a concentration of 10 µM in 0.1% FCS/DMEM for 24 h (8 + 16 h); controls were treated with the appropriate ethanol concentration (1%). Depending on subsequent experiments, cells were either lysed chemically in lysis buffer (0.1% NP-40, 0.1% Triton-X-100, 10 mM Tris, 2 mM EDTA) with or without protease inhibitor (Roche Diagnostics, Mannheim, Germany) or homogenized mechanically using a Minilys homogenizer (Peqlab, Erlangen, Germany).

### 4.3. Cell Viability

After incubation with tocopherol and tocotrienol, cell viability was identified by measuring lactate dehydrogenase activity using the Cytotoxicity Detection KitPLUS (Roche Diagnostics, Mannheim, Germany) according to the manufacturer’s instructions.

### 4.4. Cholesterol Concentration

After long-term incubation for five days (3 days with 1% FCS followed by treatment for 48 h in 0.1% FCS) with 10 µM tocopherol and tocotrienol, cholesterol content in samples was measured by using the Amplex Red Cholesterol Assay Kit (Invitrogen, Karlsruhe, Germany) according to the manufacturer’s protocol.

### 4.5. Detection of Reactive Oxygen Species

To detect ROS with aminophenyl fluorescein, cells were incubated on 96-well plates and washed once with prewarmed (37 °C) cell imaging solution (140 mM NaCl, 2.5 mM KCl, 1.8 mM CaCl_2_, 1 mM MgCl_2_, 20 mM HEPES, pH 7.4). On hundred microliters of aminophenyl fluorescein (Sigma Aldrich, Taufkirchen, Germany) in a final concentration of 10 µM in cell imaging solution were added to each well. After incubation for 60 min in the dark at 37 °C, the plate was shaken for 60 s, and fluorescence was measured with an excitation wavelength of 490 ± 10 nm and an emission wavelength of 515 ± 10 nm using a Safire^2^ Fluorometer (Tecan, Crailsheim, Germany).

To evaluate the antioxidative potential of α-tocopherol and α-tocotrienol against hydrogen peroxide, parts of the Amplex Red Cholesterol Assay Kit (Invitrogen) were used. Cells were incubated on 96-well plates with the addition of 1 µM H_2_O_2_ and washed with warmed phosphate-buffered saline. Fifty microliters 1× reaction buffer (0.1 M potassium phosphate, 50 mM NaCl, 5 mM cholic acid, 0.1% Triton X-100, pH 7.4) were added to each well, and the plate was sonificated for 10 s. Afterwards, 50 µL 1× reaction buffer containing 300 µM Amplex Red reagent and 2 U/mL horseradish peroxidase were pipetted to each sample, and the plate was shaken for 60 s. Ten microliters of 10 µM H_2_O_2_ were added to each well and mixed by shaking. The plate was incubated 40 min under light exclusion at 37 °C, shaken for another 60 s, and fluorescence was measured with an excitation wavelength of 540 ± 10 nm and an emission wavelength of 590 ± 10 nm.

### 4.6. Determination of Protein Concentration

Protein concentration in samples was determined with bicinchoninic acid as described in [79]. Before conducting the experiments, samples were adjusted to equal protein concentrations.

### 4.7. Western Blot Analysis

The total protein concentration in samples was measured and equaled as mentioned above. Sodium dodecyl sulfate polyacrylamide gel electrophoresis (SDS-PAGE) was performed with 10%–20% Tricine gradient gels (Anamed, Groß-Bieberau, Germany). Afterwards, proteins were transferred onto nitrocellulose membranes (Whatman, Dassel, Germany), and the following antibodies were used for Western blot (WB) analysis: W02 antibody (5 µg/mL) (Millipore, Billerica, MA, USA), anti-BACE1 (B0806) (1:1000, Sigma), anti-nicastrin 1 (1:500, sc25648) (Santa Cruz, Dallas, TX, USA) and anti-sAPPβ (1:250, MBS492139) (MyBioSource, San Diego, CA, USA) as primary antibodies; anti-rabbit (W401B) (1:5000, Promega, Mannheim, Germany) and anti-mouse (P0260) (1:10,000, Dako, Hamburg, Germany) as secondary antibodies.

Analysis of the Aβ protein level was performed as described earlier [80]. For detection in WB analysis, Aβ was immunoprecipitated with 20 µL protein G-Sepharose (Sigma) and W02 antibody (5 µg/mL). Aβ peptides were detected by the use of W02 in WB. The specificity, sensitivity and linearity of this assay were validated in Ida et al. [80]. Previously, we additionally confirmed the specificity, sensitivity and linearity of Aβ detection by using the W02 antibody under our experimental conditions [81].

Proteins were detected by the enhanced chemiluminescense (ECL)-method (Perkin Elmer, Rodgau-Jügesheim, Germany) and densitometrically quantified with Image Gauge V3.45 software (Fujifilm, Düsseldorf, Germany).

### 4.8. Preparation of Purified Membranes

The preparation of purified membranes was conducted according to [82]: after washing three times with precooled (4 °C) phosphate buffered saline (PBS), SH-SY5Y wt cells were scraped in sucrose buffer (10 mM Tris/HCl, pH 7.4, 1 mM EDTA, 200 mM sucrose) and homogenized using a Minilys homogenizer (Peqlab) at maximum speed for 30 s. Total protein amount was measured and equaled as mentioned above. Samples were centrifuged at 900 relative centrifugal force (rcf) for 10 min at 4 °C; supernatants were collected in a new tube and centrifuged again (Optima MAX Ultracentrifuge, Beckman Coulter, Krefeld, Germany) for 75 min at 55,000 rpm and 4 °C. Pellets were resuspended in sucrose buffer at medium speed for 10 s using Minilys (Peqlab).

### 4.9. Measurement of β- and γ-Secretase Activity

Evaluation of β- and γ-secretase activity has been described in detail earlier [20]. Shortly, fluorogenic β-secretase substrate IV (Calbiochem, Darmstadt, Germany) or γ-secretase substrate (Calbiochem) was added in a final concentration of 20 or 10 µM to samples, respectively. In the case of β-secretase, the resulting fluorescence was measured continuously under light exclusion with a Safire2 Fluorometer (Tecan) at an excitation wavelength of 345 ± 5 nm and an emission wavelength of 500 ± 2.5 nm, for γ-secretase at an excitation wavelength of 355 ± 10 nm and an emission wavelength of 440 ± 10 nm.

### 4.10. Measurement of Secretase Activity in Living Cells

SH-SY5Y wt cells were incubated with tocopherol and tocotrienol and washed with prewarmed cell imaging solution (140 mM NaCl, 5 mM KCl, 8 mM CaCl_2_, 1 mM MgCl_2_, 20 mM HEPES, pH 7.4). Thirty micromolar fluorogenic β-secretase substrate (Calbiochem) or 12 µM γ-secretase substrate (Calbiochem) in 50 µL of cell imaging solution was added, and fluorescence was determined under light exclusion at 37 °C in a Safire2 Fluorometer (Tecan) with excitation wavelengths of 345 ± 5 nm and 355 ± 10 nm and emission wavelengths of 500 ± 2.5 and 440 ± 10 nm, respectively [42].

### 4.11. Quantitative Real-Time Experiments

SH-SY5Y wt cells were incubated as mentioned above and TRIzol reagent (Invitrogen) was used to isolate total RNA, of which 2 µg were used in reverse transcription with the High-Capacity cDNA Reverse Transcription Kit (Life Technologies, Darmstadt, Germany). Fast SYBR Green Master Mix (Applied Biosystems, Darmstadt, Germany) was utilized for quantitative real-time polymerase chain reaction on a Piko Real-Time PCR System (Thermo Scientific, Waltham, MA, USA). Experiments were carried out according to the manufacturers’ protocols. Data evaluation and primer sequences are described in detail in [42].

### 4.12. Determination of Total Aβ Degradation

Evaluation of Aβ degradation has been described earlier [42]. Before incubation, N2a wt cells were cultivated for 6 h with reduced FCS (0.1%). Subsequently, tocopherol and tocotrienol (10 µM) were applied to the cells for 18 h followed by an additional treatment for 6 h with tocopherol and tocotrienol in combination with 0.5 µg/mL human synthetic Aβ40. To inhibit the activity of IDE [83], cells were additionally incubated with 10 µM human insulin (Sigma). Non-degraded human Aβ40 from cell culture supernatant was separated in SDS-PAGE and detected by Western blotting using the W02 antibody as described above.

### 4.13. Tocopherol and Tocotrienol Uptake

For quantification of α-tocopherol and α-tocotrienol in control samples, we pooled 5 independent control samples. The standard addition method with 0.02, 0.05, 0.1, 0.2, 1 and 2 nmol/mg protein of α-tocopherol and α-tocotrienol was used to calculate calibration curves (R^2^ > 0.97). The resulting control sample concentration of α-tocopherol was 0.067 ± 0.031 nmol/mg protein and for α-tocotrienol 0.034 ± 0.005 nmol/mg protein.

Tocopherols and tocotrienols were significantly (>500%, *p* ≤ 0.001) taken up by cells. Analyses were performed by mass spectrometry (see below): isotopically-labeled (±)d_6_-α-tocopherol, HPLC-grade ethyl acetate and HPLC-MS-grade acetonitrile were purchased from Sigma-Aldrich (Steinheim, Germany); 2,6-di-*tert*-butyl-4-methylphenol (butylhydroxytoluene, BHT) from Merck Schuchardt OHG (Hohenbrunn, Germany); ascorbic acid and sodium hydroxide from Carl Roth GmbH (Karlsruhe, Germany); HPLC-grade dichloromethane and HPLC-grade water from VWR International (Darmstadt, Germany); analytical reagent grade *n*-hexane from Fischer Scientific UK (Loughboroug, UK); sodium hydroxide and 37% hydrochloric acid from Merck (Darmstadt, Germany); and formic acid from AppliChem (Darmstadt, Germany).

#### 4.13.1. Sample Preparation

For extraction, we used saponification combined with liquid/liquid extraction. This combination is widely used in the literature to extract tocopherols from biological samples [48,84].

All extractions were carried out in triplicate from three independent 10-cm culture dishes, which were incubated for 24 h (8 + 16 h), as described before with 10 µM α-tocopherol, 10 µM α-tocotrienol and ethanol as the control.

Prior to extraction, the culture medium was removed; cells were washed three times with 1 mL of water and dissolved in 200 µL of water. The cells were homogenized using glass beads in Minilys (Peqlab) for 30 s at maximum speed and diluted to a protein concentration of 10 mg/mL.

The following solvents were used for extraction: (A) internal standard solution of 20 µM *d*_6_-α-tocopherol and 0.1% (*w*/*w*) BHT in acetonitrile; (B) 1.14 M ascorbic acid and 1.14 M sodium hydroxide in water/methanol 2:1 (*v*/*v*); (C) 10 M sodium hydroxide in water/methanol 2:1 (*v*/*v*); (D) 3.2 M hydrochloric acid in water/acetonitrile 2:3 (*v*/*v*); and (E) extraction solution dichloromethane/ethyl acetate/n-hexane 6:1:1 (*v*/*v*). For extraction, 30 µL of the homogenized cell sample at a 10-mg/mL protein concentration were mixed with 15 µL internal standard solution (A); 20 µL of solution (B) and 20 µL of solution (C) were added and the mixture saponificated at 55 °C under horizontal shaking conditions at 800 rpm for 20 min (Eppendorf Thermomixer, Hamburg, Germany). This was followed by cooling on ice, adding 60 µL of solution (D) and 330 µL of extraction solution (E). To achieve extraction equilibrium, the mixture was shaken for 20 min at 400 rpm, the organic layer removed, dried under a gentle stream of nitrogen and the residue dissolved in 200 µL of acetonitrile.

#### 4.13.2. LC-MS/MS

For LC-MS/MS analysis, 1 µL of the extract was injected into an Agilent (Santa Clara, CA, USA) 1200 Series capillary HPLC system. An Agilent Zorbax SB-C18 column was used for separation at a 20 µL/min isocratic flow rate of 100% acetonitrile (+0.1% formic acid). The column temperature was set to 25 °C in a MayLab (Vienna, Austria) Mistra-Switch column oven. Retention times under these conditions were 1.9 min for α-tocotrienol and 4.5 min for tocopherol. Detection of α-tocotrienol and α-tocopherol was achieved on a Sciex (Concord, ON, Canada) QTRAP 4000 triple quadrupole mass spectrometer in positive electrospray ionization (ESI) mode using selected reaction monitoring (SRM). The MS conditions were as follows: spray voltage, 5500 V; source temperature, 120 °C; curtain gas, 27 psi; Gas 1, 25 psi; Gas 2, 25 psi; entrance potential, 10 V; collision exit potential, 14 V; interface heater, on; declustering potential (DP) and collision energy (CE), compound specific. SRM-transition was *m*/*z* 423.3 → 165.1 for α-tocotrienol (CE = 27 V, DP = 55 V); *m*/*z* 429.4 → 165.1 for α-tocopherol (CE = 33 V, DP = 80 V); and *m*/*z* 435.4 → 171.1 for d_6_-α-tocopherol (CE = 33 V, DP = 80 V). For all SRM transitions, dwell-times were 200 ms. Under ESI conditions, the formation of [M − H]^+^ ions after hydride abstraction from the analyte molecules was unusual; however, a similar behavior was reported for tocopherol under atmospheric pressure chemical ionization (APCI) conditions [85]. This ion was used as the precursor ion for SRM, as it formed the base peak in the collision-induced dissociation (CID) spectra under the described conditions.

### 4.14. Statistical Analysis

Data are illustrated as an average of at least three independent experiments, instead of; the exact *n*-number for each experiment is listed in the figure legend. Error bars show the standard deviation of the mean. A two-tailed Student’s *t*-test was used for calculating statistical significance, which was set at * *p* ≤ 0.05, ** *p* ≤ 0.01 and *** *p* ≤ 0.001. For multiple comparison analysis of the different vitamin E species, post hoc ANOVA was conducted.

## 5. Conclusions

**Figure 4:**
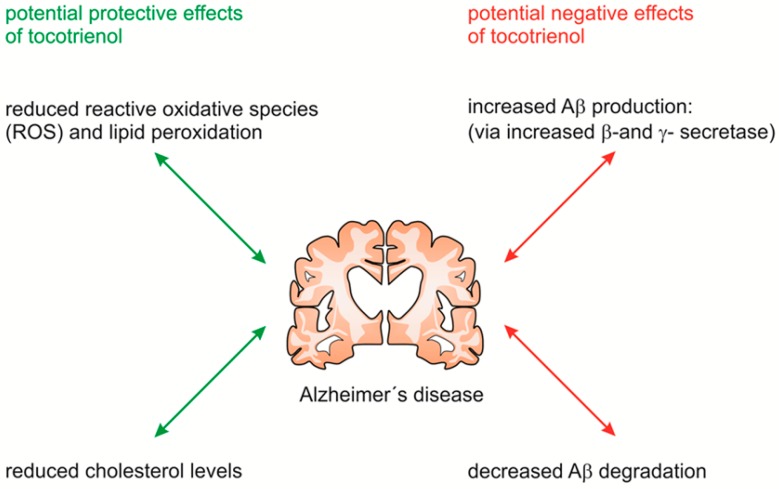
Summary of the effects of α-tocotrienol on Alzheimer’s-relevant processes. On the one hand, α-tocotrienol has positive effects by decreasing cholesterol levels and reactive oxidative, instead of; on the other hand, α-tocotrienols accelerate the amyloidogenic pathway and decrease Aβ degradation.

In summary, we could show that α-tocopherol and α-tocotrienol showed a nearly identical beneficial effect regarding the formation of ROS, whereas α-tocotrienol revealed an even more pronounced effect in the reduction of cholesterol level. Both, reduced ROS/lipid peroxidation and reduced cholesterol level are beneficial for AD (Figure 4). However, α-tocotrienol and tocopherols increase the release of Aβ by increased amyloidogenic APP processing and decrease the degradation of Aβ leading to the formation of neuritic plaques. Indisputable positive effects, but also negative modes of action of the vitamin E family with respect to the mechanisms involved in AD pathogenesis have to be taken into consideration when deciding whether patients could benefit from vitamin E supplementation or not.

## Supplementary Materials

Supplementary materials can be found at www.mdpi.com/1422-0067/17/11/1809/s1.
