# Supplementary material for: Tocotrienol Affects Oxidative Stress, Cholesterol Homeostasis and the Amyloidogenic Pathway in Neuroblastoma Cells: Consequences for Alzheimer’s Disease
Source: Int J Mol Sci. 2016 Oct 29;17(11):1809. doi: 10.3390/ijms17111809 (PMC5133810; doi:10.3390/ijms17111809)
Supplement: Supplementary file 1 [file ijms-17-01809-s001.pdf]

Supplementary Materials: Tocotrienol Affects Oxidative Stress, Cholesterol Homeostasis and the Amyloidogenic Pathway in Neuroblastoma Cells: Consequences for Alzheimer's Disease

Marcus O. W. Grimm, Liesa Regner, Janine Mett, Christoph P. Stahlmann, Pascal Schorr, Christopher Nelke, Olga Streidenberger, Hannah Stoetzel, Jakob Winkler, Shatha R. Zaidan, Andrea Thiel, Kristina Endres, Heike S. Grimm, Dietrich A. Volmer and Tobias Hartmann

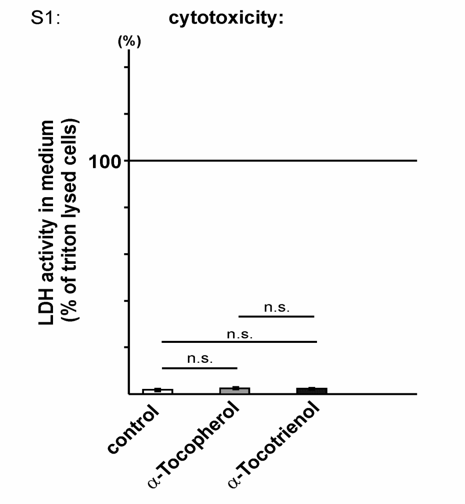

Figure S1. No significant effect of α-tocopherol and α-tocotrienol on cell viability measured by LDH activity in medium (cytotoxicity solvent control: 1.8% ± 0.1%; cytotoxicity α-tocopherol: 2.5% ± 0.3%, p = 0.22 compared to the solvent, n = 4; cytotoxicity α-tocotrienol: 2.3% ± 0.2%, p = 0.41 compared to the solvent, p = 0.86 compared to α-tocopherol, n = 4). Statistical significance as described for Figure 1 (n.s., not significant).

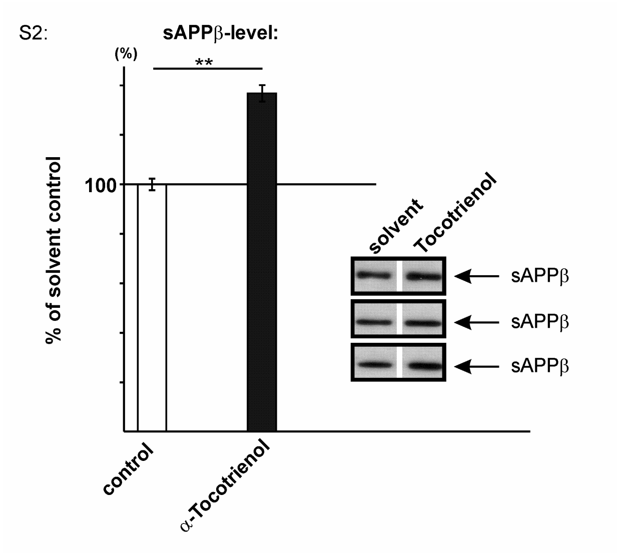

Figure S2. Increased secretion of sAPPβ (136.8% ± 3.3%, p = 0.004, n = 3) by SH-SY5Y APP695 transfected cells after incubation with α-tocotrienol. Statistical significance as described for Figure 3 (** $p \leqslant 0.01$).

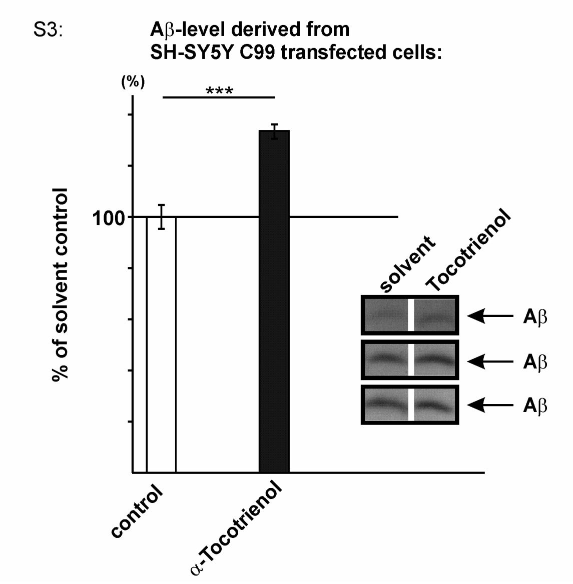

Figure S3. Enhanced production of Aβ (133.6% ± 2.8%, $p \leq 0.001$, $n = 12$) by SH-SY5Y C99 transfected cells after incubation with α-tocotrienol. 100% in the control cells corresponds to 10.0 ng/mL total Aβ. Statistical significance as described for Figure 3 (*** $p \leqslant 0.001$).

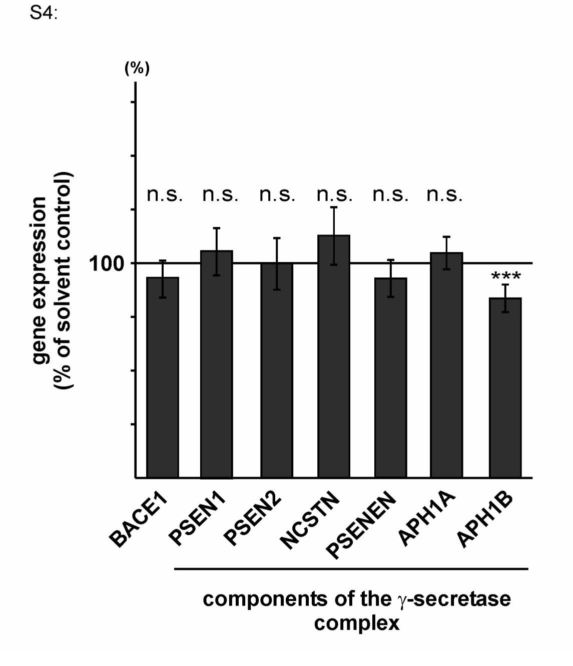

Figure S4. Unchanged gene expression of the components of the β- and γ-secretase measured by real-time PCR. BACE1 = β-site APP cleaving enzyme 1: 93.2% ± 4.3%, $p = 0.140$, $n = 8$; PSEN1 = presenilin 1: 107.9% ± 5.5%, $p = 0.17$, $n = 9$; PSEN2 = presenilin 2: 102.1% ± 6.0%, $p = 0.73$, $n = 9$; NCSTN = nicastrin: 110.4% ± 6.6%, $p = 0.13$, $n = 9$; PSENEN = presenilin-enhancer 2: 95.7% ± 5.2%, $p = 0.42$, $n = 9$; APH1a = anterior-pharynx-defective 1 a: 105.4% ± 3.8%, $p = 0.18$, $n = 9$; APH1b = anterior-pharynx-defective 1 b: 85.2% ± 3.4%, $p \leq 0.001$, $n = 9$). Statistical significance as described for Figure 3 (*** $p \leqslant 0.001$; n.s., not significant).

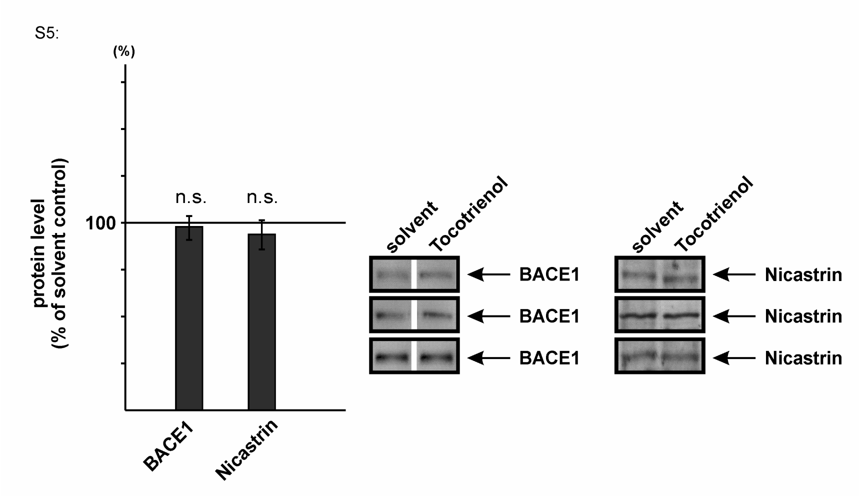

Figure S5. Unaltered protein level of BACE1 (97.8% ± 3.2%, p = 0.68, n = 7) and nicastrin (91.8% ± 3.9%, p = 0.13, n = 4) in the presence of α-tocotrienol. Statistical significance as described for Figure 3 (n.s., not significant).
